# Reconstruction of cytosolic fumaric acid biosynthetic pathways in *Saccharomyces cerevisiae*

**DOI:** 10.1186/1475-2859-11-24

**Published:** 2012-02-15

**Authors:** Guoqiang Xu, Liming Liu, Jian Chen

**Affiliations:** 1 State Key Laboratory of Food Science and Technology, Jiangnan University, Wuxi 214122, China; 2 The Key Laboratory of Industrial Biotechnology, Ministry of Education, School of Biotechnology, Jiangnan University, Wuxi 214122, China; 3 The Key Laboratory of Carbohydrate Chemistry and Biotechnology, Ministry of Education, School of Biotechnology, Jiangnan University, Wuxi 214122, China

**Keywords:** Fumaric acid, *Saccharomyces cerevisiae*, *Rhizopus oryzae*, *RoMDH*, *RoFUM1*, *PYC2*

## Abstract

**Background:**

Fumaric acid is a commercially important component of foodstuffs, pharmaceuticals and industrial materials, yet the current methods of production are unsustainable and ecologically destructive.

**Results:**

In this study, the fumarate biosynthetic pathway involving reductive reactions of the tricarboxylic acid cycle was exogenously introduced in *S. cerevisiae *by a series of simple genetic modifications. First, the *Rhizopus oryzae *genes for malate dehydrogenase (*RoMDH*) and fumarase (*RoFUM1*) were heterologously expressed. Then, expression of the endogenous pyruvate carboxylase (*PYC2*) was up-regulated. The resultant yeast strain, FMME-001 ↑*PYC2 *+ ↑*RoMDH*, was capable of producing significantly higher yields of fumarate in the glucose medium (3.18 ± 0.15 g liter^-1^) than the control strain FMME-001 empty vector.

**Conclusions:**

The results presented here provide a novel strategy for fumarate biosynthesis, which represents an important advancement in producing high yields of fumarate in a sustainable and ecologically-friendly manner.

## Background

Fumaric acid, a four-carbon dicarboxylic acid, is widely used in modern day industries ranging from materials to human and animal food and therapeutic drugs. Its abilities to be converted into pharmaceutical products and act as starting material for polymerization and esterification reactions have led to the U.S. Department of Energy to designate fumaric acid among the top 12 biomass building-block chemicals with potential to significantly enhance the economy [1]. Fumaric acid is currently produced in large scale by one of three different routes: (*i*) chemical synthesis; (*ii*) enzymatic catalysis; and (*iii*) fermentation. The process of chemical synthesis requires heavy metal catalysts, organic solvents, high temperature and high pressures [2], which makes the conversion of maleic anhydride to fumarate can be ecologically destructive. Enzymatic conversion of maleic anhydride derived from petroleum into fumarate is unsustainable and costly due to the dwindling global supply of petroleum resources and increasing oil prices, despite the fact that a high conversion yield is achievable [3]. A fermentation process based on fungi, such as *Rhizopus oryzae *and *Rhizopus arrhizus*, has been successfully used for fumaric acid production [4]; however, this process is limited on the industrial scale since these fungi are difficult to grow and their morphology can strongly affect production characteristics. Moreover, since these fungi harbor potentially pathogenic properties, product safety is questionable.

The yeast *Saccharomyces cerevisiae *is a well-established industrial production organism, and is especially known for its outstanding capacity to produce ethanol. This yeast species also possesses good cultivation characteristics, including requiring a simple chemically defined medium, being fairly resistant to inhibitors that are normally present in biomass hydrolysates, and having an extraordinarily robust tolerance for high sugar and ethanol concentrations. In addition, *S. cerevisiae's *robust tolerance towards acidic conditions represents a major advantage in that it lowers the risk of contamination in industrial fermentation [5]. It is believed that the long history of the safe usage in the food and beverage industry may facilitate and expedite of *S*. *cerevisiae'*s federal approval for use in the production of organic acids destined for human consumption.

In addition, this type of yeast is a popular eukaryotic model organism for the study of fundamental biological processes. Its genome has been completely sequenced, and its genetic and physiologic properties are not only well-characterized but established tools of genetic manipulation and screening research strategies [6]. Several databases such as the Saccharomyces Genome Database (SGD) (http://www.yeastgenome.org), have provided an enormous amount of information on *S. cerevisiae *genes, open reading frames, and gene products. Likewise, a multitude of technologies have been developed for high-throughput analysis of the yeast transcriptome, proteome, metabolome, and interactome [7]. Collectively, these features have made yeast a very attractive platform for metabolic engineering. In particular, *S. cerevisiae *is being investigated for its capacity for large-scale biotechnological production of organic acids. Indeed, some progress has been made in exploring the utility of metabolic engineering of *S. cerevisiae*, and it has been successfully manipulated to produce monocarboxylic acid pyruvate [8], lactate [9], dicarboxylic acid malate [10,11], and succinate [2].

Despite these advances, metabolic engineering of *S. cerevisiae *for the production of biotechnologically interesting carboxylic acids from renewable feedstocks remains to be optimized [12]. *S. cerevisiae *in its natural state cannot accumulate large amounts of fumarate in the cytosol, due to the fact that cytosolic fumarase catalyzes the conversion of fumarate to L-malate but not *vice versa *[13]. Stein and colleagues have demonstrated that a single translation product of the *FUM1 *gene that encodes fumarase is distributed between the cytosol and mitochondria in *S. cerevisiae *[14]. Many studies have since concentrated on elucidating the mechanism underlying this post-translation distribution profile [15-18]. Surprisingly, no reports in the literature have described attempts to up-regulate fumarase via metabolic engineering approaches in order to increase fumarate accumulation in *S. cerevisiae*.

**Figure 1:**
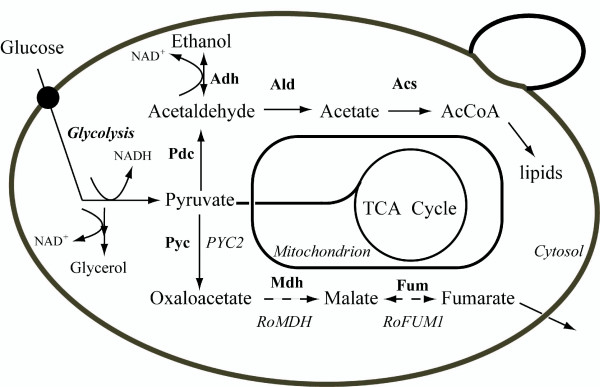
**The cytosolic fumarate biosynthetic pathway in *S. cerevisiae***. Abbreviations of enzymes: Pyc, pyruvate carboxylase; Mdh, malate dehydrogenase; Fum, fumarase; Pdc, pyruvate decarboxylase; Adh, alcohol dehydrogenase; Ald, acetaldehyde dehydrogenase; Acs, acetyl-CoA synthetase.

We considered that fumarate can be accumulated and excreted by *R. oryzae *through reductive reactions of the tricarboxylic acid cycle [19,20], and asked whether *S. cerevisiae *can accumulate fumarate by a completely cytosolic fumarate biosynthetic pathway. Therefore, we sought to develop a novel metabolic pathway for fumarate production in *S. cerevisiae *by introducing the follow genetic improvements (Figure 1): (*i*) heterologous expression of cytosolic *R. oryzae *malate dehydrogenase; (*ii*) high-level expression of the *R. oryzae *fumarase; and (*iii*) over-expression of the native cytosolic pyruvate carboxylase encoded by *PYC2*. In addition, we evaluated which protein represents the limiting factor for fumarate formation.

## Results

### Comparative analysis of two species malate dehydrogenases on fumarate production capabilities

**Figure 2:**
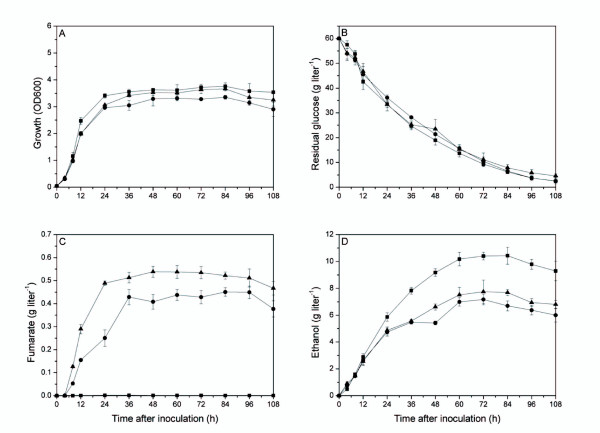
**Fermentation profile for cell growth, glucose utilization and product accumulation during aerobic batch cultures of control strain FMME-001 empty vector, engineered strains FMME-001 ↑*MDH3ΔSKL *and FMME-001 ↑*RoMDH *with 6% glucose**. A: growth, B: residual glucose, C: fumaric acid and D: ethanol. Symbols: square, FMME-001 empty vector, circle, FMME-001 ↑*MDH3ΔSKL*, triangle, FMME-001 ↑*RoMDH*. Error bars indicate standard deviation (n = 3).

Over-expression of malate dehydrogenase in the cytosol of *S. cerevisiae *was achieved by two methods. In the first, endogenous malate dehydrogenase was overexpressed. One of the three malate dehydrogenase isoenzymes in *S. cerevisiae*, Mdh2p, is known to be subject to glucose catabolite inactivation [21] even though it is located in the cytosol, ultimately limiting batch cultivation on glucose. Therefore, the strategy selected for cytosolic malate dehydrogenase overexpression was based on re-targeting Mdh3 by removing the C-terminal SKL tripeptide [22]. In the second, the heterologous *RoMDH *gene was over-expressed. The corresponding engineered strains, FMME-001 ↑*MDH3ΔSKL *and FMME-001 ↑*RoMDH*, were evaluated for the titers of fumarate produced. FMME-001 ↑*MDH3ΔSKL *achieved an average of 0.44 ± 0.03 g liter^-1^, while FMME-001 ↑*RoMDH *achieved an average of 0.54 ± 0.04 g liter^-1 ^(Figure 2). These results indicated that the FMME-001 ↑*RoMDH *strain had a 22.7% higher fumarate yield than the strain FMME-001 ↑*MDH3ΔSKL *strain. Moreover, the higher Y_P/S _value associated with the FMME-001 ↑*RoMDH *strain led us to focus our subsequent studies on the cytosolic malate dehydrogenase encoded by the *RoMDH*.

### Fumaric acid characterization

**Figure 3:**
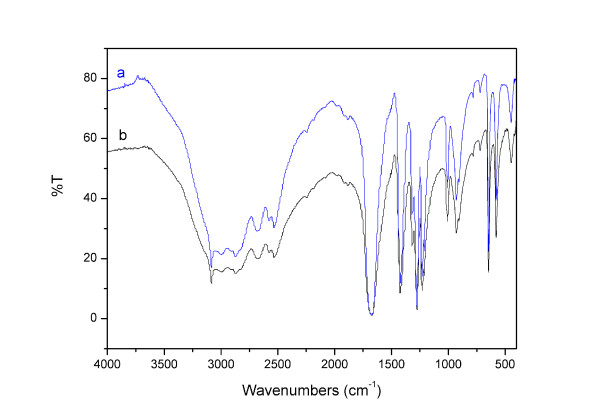
**The IR spectra of fumaric acid**. (a) sample, (b) the fumaric acid standard.

**Figure 4:**
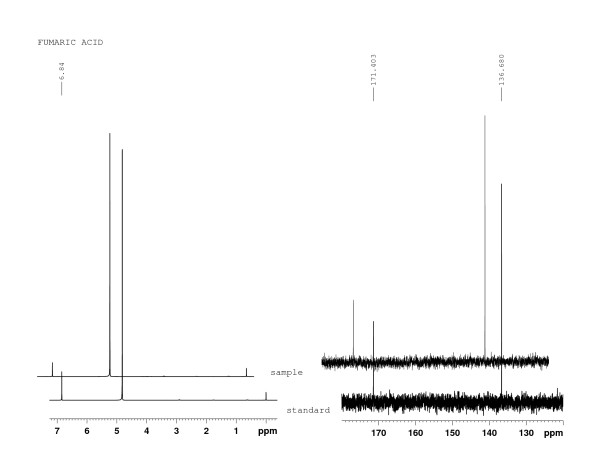
^**1**^**H NMR and ^13^C NMR spectra of sample from engineered strain and the fumaric acid standard**.

The Fourier transform infrared (FT-IR) spectra of sample isolated from the fermentation broth of our engineered strain was consistent with the fumaric acid standard (Figure 3). In addition, the ^1^H NMR spectra and ^13^C NMR spectra showed excellent matches between the sample and fumaric acid standard (Figure 4). Thus, these results confirmed that fumaric acid was, in fact, synthesized by our engineered *S. cerevisiae *strain.

### Effects of *RoMDH *and *RoFUM1 *co-expression on fumarate production

In order to augment the fumarate synthesis ability of FMME-001 ↑*RoMDH*, the *R. oryzae RoFUM1 *gene encoding cytosolic fumarase was simultaneously over-expressed from a high copy vector. However, the co-expression strain FMME-001 ↑*RoMDH *+ ↑*RoFUM1 *did not exhibit a significantly higher titer of fumarate as expected; instead, the titer decreased to 0.38 ± 0.03 g liter^-1^, and slightly more malic acid, citric acid, and succinic acid were detected (Table 1).

**Table 1:** Shake flask cultivation characteristics of cell growth, glucose utilization, and production of fumarate, pyruvate, ethanol, glycerol and some other metabolites of the TCA cycle

Strain	FMME-001 empty vector	FMME-001 ↑*RoMDH*	FMME-001 ↑*RoMDH*+↑*RoFUM1*	FMME-001 ↑*PYC2 *+ ↑*RoMDH*
Glucose consumption (g liter^-1^)	48	48	48	48
Biomass (OD600)	3.58 (± 0.28)	3.23 (± 0.22)	2.98 (± 0.14)	3.70 (± 0.19)
Ethanol (g liter^-1^)	10.40 (± 0.35)	7.74 (± 0.43)	7.41 (± 0.37)	6.53 (± 0.63)
Glycerol (g liter^-1^)	1.58 (± 0.12)	1.40 (± 0.13)	1.34 (± 0.12)	1.65 (± 0.14)
Pyruvic acid (g liter^-1^)	0.32 (± 0.015)	0.42 (± 0.018)	0.41 (± 0.056)	0.26 (± 0.011)
Fumaric acid (g liter^-1^)	< 0.00 (± 0.00)	0.54 (± 0.04)	0.38 (± 0.03)	3.18 (± 0.15)
Malic acid (g liter^-1^)	0.18 (± 0.02)	0.72 (± 0.06)	0.80 (± 0.06)	0.66 (± 0.07)
Citric acid (g liter^-1^)	0.056 (± 0.003)	0.068 (± 0.005)	0.076 (± 0.004)	0.057 (± 0.003)
α-Ketoglutarate (g liter^-1^)	0.027 (± 0.002)	0.033 (± 0.003)	0.030 (± 0.002)	0.064 (± 0.008)
Succinic acid (g liter^-1^)	0.016 (± 0.002)	0.012 (± 0.002)	0.018 (± 0.003)	0.060 (± 0.004)

### Fumarate synthesis pathway enzyme activities and transcriptional levels

**Figure 5:**
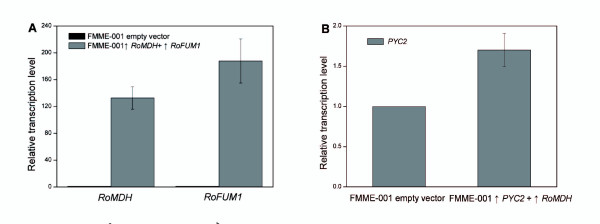
**Relative gene expression levels.** (A) *RoMDH *and *RoFUM1 *in the mutant strain FMME-001↑*RoMDH *+ ↑*RoFUM1 *and the parent strain FMME-001 empty vector. Relative gene expression level of (B) *PYC2 *in the mutant strain FMME-001↑*PYC2 *+ ↑*RoMDH *and the parent strain FMME-001 empty vector. Relative transcription levels were normalized to the transcription level of the β*-ACTIN *gene, which was taken as 1. The presented values are averages of three independent experiments; the error bars indicate standard deviations.

To elucidate the molecular mechanism underlying the low titer of fumarate accumulation in the cytosol of the engineered yeast strain FMME-001 ↑*RoMDH *+ ↑*RoFUM1*, the activity and transcriptional level of key enzymes in the fumarate synthesis pathway were determined. The specific activity of malate dehydrogenase was found to be 40.5 ± 4.3 U min^-1 ^(mg protein)^-1^, approximately 18-fold higher than that of the control strain (2.2 ± 0.31 Umin^-1 ^(mg protein)^-1^). In addition, the specific activity of fumarase was only 0.030 ± 0.002 U min^-1 ^(mg protein)^-1^, while the activity of the reverse reaction was 0.140 ± 0.010 U min^-1 ^(mg protein)^-1^. Pyruvate carboxylase activity of FMME-001 ↑*RoMDH *+ ↑*RoFUM1 *was only 0.0022 ± 0.0002 U min^-1 ^(mg protein)^-1 ^(Table 2). The gene expression levels of *RoMDH *and *RoFUM1 *were dramatically increased, as detected by quantitative real-time PCR (QT-PCR) (Figure 5A). Taken together, these results suggest that pyruvate carboxylase represents the rate limiting factor of fumarate production.

**Table 2:** Comparison of the activities of key enzymes activities in the control strain and engineered strain

Strain^*a*^	Enzyme activity^*b *^(U min^-1 ^(mg protein)^-1^) of:			
	
	MDH	FUM, malate to fumarate	FUM, fumarate to malate	PYC
Reference	2.2 ± 0.31	0.002 ± 0.000	0.021 ± 0.002	0.0022 ± 0.0002
FMME-001 ↑*RoMDH *+ ↑*RoFUM1*	40.5 ± 4.3	0.030 ± 0.002	0.140 ± 0.010	0.0022 ± 0.0002
Ratio	18.41	15.0	6.67	1.00

^*a*^Yeast strains were grown in shake flasks on glucose medium, unless otherwise indicated in the text.

^*b*^MDH, malate dehydrogenase; FUM, fumarase; PYC, pyruvate carboxylase. Errors represent deviations from the means (for each condition, n = 3).

### Effects of over-expression of endogenous pyruvate carboxylase

The integration-expression of *PYC2 *in the engineered strain FMME-001 ↑*RoMDH *resulted in increased specific activity of pyruvate carboxylase by 20.8% (*P *< 0.05, n = 3). Consequently, the fumarate titer also increased by a remarkable 488.9% (from 0.54 ± 0.04 g liter^-1 ^to 3.18 ± 0.15 g liter^-1^), as compared to the control strain FMME-001 (Table 1).

## Discussion

*S. cerevisiae *is an important industrial tool and biological model. Although this particular yeast species does not typically excrete a high titer of organic acids, its high metabolic capacity makes it a potential promising platform for engineered organic acid production. In order to establish this type of system, however, heterologous metabolic pathways must be introduced and optimized to facilitate the conversion of sugars to organic acids. The study described herein set about to accomplish the heterologous introduction of a cytosolic fumarate biosynthetic pathway into *S. cerevisiae *via genetic engineering; ultimately, this simple manipulation achieved 3.18 ± 0.15 g liter^-1 ^fumarate production. Indeed, the level of fumarate achieved by this engineered strain was low as compared with that from *Rhizopus oryzae *or *Rhizopus arrhizus*. The most likely reasons for this, and focus of our future studies to improve the *S. cerevisiae*-based method, are as follows:

A novel metabolic pathway for fumarate production was established in *S. cerevisiae*; this series of chemical reactions began with carboxylation of pyruvate to oxaloacetate, followed by reduction to malate, and finally reduction to fumarate (Figure 1). Flux through the cytosolic fumarate biosynthetic pathway is principally determined by the abundance and biochemical properties of three enzymes in the fumarate biosynthetic pathway, transporters, and regulatory proteins, as well as their interactions with each other and the metabolites generated by each.

A previous study had demonstrated that over-expression of a truncated form of *MDH3 *(*MDH3ΔSKL*), which remained sequestered in the cytosol, led to a more than 20-fold increase in cytosolic malate dehydrogenase activity [10]. Meanwhile, an unrelated study found that *R. oryze *had naturally occurring high activities of cytosolic malate dehydrogenase (encoded by the gene *RoMDH*) corresponding with fumarate production [19]. Furthermore, a higher titer and yield of fumarate was achieved when *RoMDH *was overexpressed in *S. cerevisiae*. Together, these findings indicated that the cytosolic malate dehydrogenase encoded by the *RoMDH *gene was a potential candidate for further biological manipulation to enhance fumarate production. Moreover, a relatively higher activity of fumarase was reported in *R. oryzae *[19,20]. Contrary to expectations, however, when *RoFUM1 *was heterologously expressed in *S. cerevisiae*, the fumarate titer decreased and the malate titer increased, presumably due to the fact that the *RoFUM1*-encoded fumarase has about 4.5-fold higher affinity for fumarate than for L-malate [19]. Subsequently, our evaluations of the enzyme activity revealed that the malate dehydrogenase activity was significantly increased in the engineered strain, as compared to that in the parent strain, and only very low levels of pyruvate carboxylase were detectable in the engineered strain FMME-001 ↑*RoMDH*. Furthermore, comparative analysis of the *RoMDH *and *RoFUM1 *transcripts demonstrated that both genes were significantly up-regulated. Collectively, these results suggest that pyruvate carboxylase has a relatively low degree of control over the rate of fumarate production in the engineered strain FMME-001 ↑*RoMDH*.

With the integrated-expression of the *PYC*2 gene in *S. cerevisiae *the titer of fumarate increased from 0.54 ± 0.02 g liter^-1 ^to 3.18 ± 0.15 g liter^-1^. Quantitative real-time PCR analysis showed that a small increase in the transcriptional level of *PYC2 *had occurred after *PYC2 *integrated-expression (70.2%, *P *< 0.05, n = 3) (Figure 5B). This result indicated that pyruvate carboxylase is able to control the carbon flux conduit towards fumarate in the engineered strain. This finding was consistent with the hypothesis that C4 dicarboxylic acids require large fluxes through the carboxylating anaplerotic pathways to achieve particularly high yield [12]. Furthermore, over-expression of the native *PYC2 *gene in *S. cerevisiae *is known to elicit a higher specific malate production due to increased carboxylation of pyruvate to oxaloacetate [10].

In this study, 6.53 ± 0.63 g liter^-1 ^ethanol and 1.65 ± 0.14 g liter^-1 ^glycerol were detected in the fermentation broth of FMME-001 ↑*PYC2 *+ ↑*RoMDH *(Table 1). This phenomenon is known as "overflow metabolism". In *S. cerevisiae*, overflow metabolism begins when the specific glucose uptake rate (or the glycolytic flux) exceeds a threshold rate, resulting in the formation of ethanol and glycerol [23]. Thus, in order to further increase fumarate accumulation it is necessary to develop a metabolic-engineering strategy to reduce or eliminate ethanol formation.

This goal can be achieved by disrupting or weakening the specific enzymes, such as pyruvate decarboxylase and alcohol dehydrogenase [8,24], or by manipulating the available concentration of thiamine that is required by pyruvate decarboxylase [25]. Meanwhile, increasing the direct oxidation of NADH, either by enhancing respiration via improvements in the dissolved oxygen content or by overexpressing an alternative oxidase [23], is also an effective approach to reduce ethanol production.

## Conclusions

In this study, we sought to explore the feasibility of *S. cerevisiae *as a metabolically-engineered platform to safely and effectively produce high yields of fumarate by using a completely cytosolic fumarate biosynthetic pathway. Previous studies had indicated that cytosolic malate dehydrogenase encoded by the *RoMDH *gene is more effective than that encoded by the *MDH3ΔSKL *gene, and that pyruvate carboxylase represents the rate limiting factor of fumarate production. To the best of our knowledge, the study described herein is the first to demonstrate *S. cerevisiae *as a useful host strain for synthesizing fumarate by introducing genes from *R. oryzae*. This study not only provides a novel and environmentally-friendly method of producing fumaric acid, but also suggests strategies to further improve the fumarate yield in future researches.

## Methods

### Yeast strains and maintenance

All yeast strains used in this study were derived from *S. cerevisiae *BMA64 (MATa/MATα ura3-52/ura3-52; trp1Δ 2/trp1Δ 2; leu2-3,112/leu2-3,112; his3-11/his3-11; ade2-1/ade2-1; can1-100/can1-100; from Euroscarf, Frankfurt, Germany) (Table 3). Stock cultures were prepared by adding glycerol (25% vol/vol) to shake flask cultures (detailed below); aliquots (1 ml) were placed in sterile vials and stored at -80°C until use.

**Table 3:** Plasmids and strains used in this study

Plasmids or strains	Genotype or description	Source or reference
Plasmids		
pY26 TEF/GPD	2 μm *URA3*, P_GPD _/T_CYC1_, P_TEF _/T_ADH1_	Lab collection
pRS305TEF	integration vector *LEU2*, P_TEF1 _/T_ADH1_	[26]
*S. cerevisiae *strains		
BMA64	*MATa/MATα*; reference strain	Euroscarf
FMME-001 empty vector	*MATa/MATα *{pY26TEF/GPD}	This study
FMME-001 ↑*MDH3ΔSKL*	*MATa/MATα *{pY26TEF/GPD-*MDH3ΔSKL*}	This study
FMME-001 ↑*RoMDH*	*MATa/MATα *{pY26TEF/GPD-*RoMDH*}	This study
FMME-001 ↑*RoMDH *+ ↑*RoFUM1*	*MATa/MATα *{pY26TEF-*RoFUM1*/GPD-*RoMDH*}	This study
FMME-001 ↑*PYC2 *+ ↑*RoMDH*	*MATa/MATα *{pRS305TEF1-*PYC2*, pY26TEF/GPD- *RoMDH*}	This study

### Isolation of the fumarate--biosynthesis genes

*R. oryzae *NRRL1526 (ATCC 10260) was cultured in fermentation medium until the acid production phase was reached, then the fungus was harvested by centrifugation and snap frozen in liquid nitrogen for storage at -80°C until use. Total RNA was extracted by RNAprep pure Plant Kit (Tiangen Biotech Co., Ltd., Beijing, China), according to the manufacturer's protocol. Poly(A)^+ ^mRNA was purified from total RNA using Oligo(dT)15 primer in accordance with the manufacturer's instruction, then mRNA were used for cDNA synthesis by means of the Quantscript RT Kit (Tiangen Biotech Co., Ltd.).

The PCR primer pairs covering the entire open reading frame (ORF) of these two genes were designed according to the GenBank sequences of *R. oryzae *using the Primer Premier v5.0 software (Table 4). Thermal cycling parameters comprised an initial denaturation step at 94°C for 5 min, followed by 29 cycles of denaturation at 94°C for 30 s, annealing at 52°C for 30 s, and extension at 72°C for 1 min/kb, with a final single extension step at 72°C for 10 min. The reactions were carried out in a C1000™ Thermal Cycler instrument (Bio-Rad, USA). Thereafter, a 1485-bp fragment of the *RoFUM1 *gene and a 1014-bp fragment of the *RoMDH *gene were amplified by nested PCR. The sequences of these two gene fragments were submitted to GenBank under accession numbers HM130701 (*RoFUM1*) and HM130702.1 (*RoMDH*), and their nucleotide sequence alignments showed high identity (99.7% for *RoFUM1*; 95.6% for *RoMDH*).

**Table 4:** Primers used in this study for gene cloning and plasmid construction

Oligonucleotides	Sequences, 5'-3'	Usage
F-*RoFUM1*	ATGTTGCGAGCTTCTGCTACC	Cloning of *RoFUM1*
R-*RoFUM1*	TTAATCCTTGGCAGAGATCATATCTT	
F-*RoMDH*	ATGTTTGCCGCCTCTCGTG	Cloning of *RoMDH*
R-*RoMDH*	TTATTGAACAAAGCTGTTACCCTTG	
BamHI-F(*RoMDH*)	CGGGATCCATGTTTGCCGCCTCTCGTG	Construction of pY26TEF-GPD-*RoMDH*
HindIII-R(*RoMDH*)	CCCAAGCTTTTATTGAACAAAGCTGTTACCCTTG	
BamHI-F(*MDH3ΔSKL*)	CGGGATCCATGGTCAAAGTCGCAATTCTTG	Construction of pY26TEF-GPD- *MDH3ΔSKL*
HindIII-R(*MDH3ΔSKL*)	CCCAAGCTTTCAAGAGTCTAGGATGAAACTCTTGCCT	
NotI-F(*RoFUM1*)	ATAAGAATGCGGCCGCATGTTGCGAGCTTCTGCTACC	Construction of pY26TEF- *RoFUM1-*GPD- *RoMDH*
BglII-R(*RoFUM1*)	GAAGATCTTTAATCCTTGGCAGAGATCATATCTT	
PstI -F(*PYC2*)	AACTGCAGATGAGCAGTAGCAAGAAATTGGC	Construction of pRS305TEF1-*PYC2*
SalI -R(*PYC2*)	ACGCGTCGACTTACTTTTTTTGGGATGGGGGT	

*Abbreviations*: F, forward; R, reverse. Underlined sequences indicate the recognition sites for the indicated restriction enzymes.

### Construction of the plasmids

The plasmids used in this study are listed in Table 3. Gene-specific primers (Table 4) were designed to amplify *RoMDH, RoFUM, MDH3ΔSKL *and *PYC2*. The *RoMDH *and *RoFUM1 *genes were amplified by PCR using the cDNA of *R. oryzae *NRRL1526 as template. Both the resultant PCR fragment of *RoMDH *and expression vector pY26TEF-GPD were digested with BamHI and HindIII sites and ligated together to create pY26TEF-GPD-*RoMDH*. Then, the PCR product of *RoFUM1 *and pY26TEF-GPD-*RoMDH *were digested with NotI and BglII and ligated together to create the pY26TEF-*RoFUM1*-GPD-*RoMDH *plasmid.

The *S. cerevisiae MDH3ΔSKL *gene was amplified by PCR from chromosomal DNA of BMA64-1A (MATa *leu2-3,112 his3-11,15 trp1Δ can1-100 ade2-1 ura3-1*) using the primers BamHI-F(*MDH3ΔSKL*) and HindIII-R(*MDH3ΔSKL*). The resulting fragment contains the entire *MDH3 *gene minus the last 9 base pairs that encode the peroxisomal targeting sequence (the tripeptide SKL). The PCR fragment and pY26TEF-GPD vector were digested with BamHI and SalI and ligated to create pY26TEF-GPD-*MDH3ΔSKL*. The gene coding for *S. cerevisiae *pyruvate carboxylase, *PYC2*, was also amplified by PCR from chromosomal DNA of BMA64-1A but using the primers PstI-F(*PYC2*) and SalI-R(*PYC2*). The PCR fragment and pRS305TEF1 vector were digested with PstI and SalI and ligated to create pRS305TEF1-*PYC2*.

### Yeast transformation

DNA was introduced into yeast cells using a Frozen-EZ Yeast Transformation II kit (Zymo Research, Orange, CA, USA), according to the recommended protocol. The transformants were selected on agar plates of Synthetic Complete (SC) Selection medium lacking specific amino acid or pyrimidine for the auxotrophic markers.

### Shake flask cultivation

Shake flask cultures were grown on fermentation medium containing (per liter): demineralized water, 60 g glucose, 2 g CO (NH_2_)_2_, 5 g KH_2_PO_4_, and 0.8 g MgSO_4_·7H_2_O. CaCO_3 _(dry-heat sterilized at 160°C for 30 min) was used as a pH buffer of the medium. Prior to use, the medium pH was set to 5.5 with NaOH and heat sterilized for 20 min at 115°C. After cooling, the corresponding filter-sterilized amino acid mix, uracil, and adenine were added. The shake flask technique was performed at 30°C in an orbital shaker at 200 rpm. The seed medium consisted of (per liter): 20 g glucose, 1.7 g Yeast Nitrogen Base (without amino acids or ammonium sulfate), and 5 g (NH_4_)_2_SO_4_. The medium pH was adjusted to 5.5 with NaOH. The seed culture was inoculated with well-grown yeast on an agar slant and incubated for 24 h in a 250 ml flask containing 20 ml seed medium. Then, the broth was centrifuged, the pellet was resuspended in isometric fresh fermentation medium, and the cell suspension was inoculated into a 250 ml shake flask containing 50 ml fermentation medium. All experiments were carried out in triplicate.

### Metabolite analysis

Cell growth was determined by measuring the OD_600 _after desired dilution. Extracellular concentrations of fumarate, ethanol, glycerol and glucose were determined by high performance liquid chromatography, using an Aminex HPX-87H column (Bio-Rad, Hercules, CA, USA) eluted with 0.0275% (v/v) H_2_SO_4 _at a flow rate of 0.6 ml min^-1 ^at 35°C. Fumarate was detected with Agilent (Santa Clara, CA, USA) 1100 series VWD detector at 210 nm. Ethanol, glycerol and glucose were detected with an 1100 series Agilent refractive index detector.

### Confirmation of fumaric acid biosynthesis by FT-IR, ^1^H NMR, and ^13^C NMR

Cell cultures of the engineered strain were centrifuged and the harvested supernatent adjusted to pH 1.0 by addition of HCl. Following acidification, the fumaric acid precipitated out of the solution and was recovered by drying in a rotary dryer. The obtained sample was processed, along with the fumaric acid standard sample, for FT-IR, ^1^H NMR, and ^13^C NMR analyses. The FT-IR spectra were recorded on a Nicolet Nexus 470 spectrophotometer with a DTGS detector. ^1^H NMR (400 MHz, D_2_O, 25°C) and ^13^C NMR (100 MHz, D_2_O, 25°C) spectra were recorded on an Avance III 400 MHz digital NMR spectrometer.

### Enzyme assays

The strains were cultivated in fermentation medium containing 60 g liter^-1 ^glucose. The assay mixture for malate dehydrogenase contained 0.1 M potassium phosphate buffer (pH 8.0) and 0.15 mM NADH in demineralized water. The reaction was started by the addition of 1 mM oxaloacetate. Malate dehydrogenase activity was measured spectrophotometrically by monitoring NADH oxidation at 340 nm.

The pyruvate carboxylase activity was measured by the method described by de Jong-Gubbels, P [27]. The reaction mixture (1 ml) contained 100 μmol Tris buffer (pH 7.8), 7.5 μmol MgSO_4_, 0.1 μmol acetyl-CoA, 20 μmol KHCO_3_, 0.15 μmol NADH, 12 U malate dehydrogenase (Sigma), 10 μmol potassium pyruvate, and cell-free extract. The reaction was started with addition of 4 μmol ATP. The activity was measured at 340 nm.

The fumarase activity produced with L-malic acid as the substrate was determined by measuring L-malic acid (50 mM) consumption at 250 nm [28]. The fumarase activity with fumaric acid as the substrate was assayed under the same conditions and by following the decrease in absorbance at 300 nm that occurs when fumaric acid is converted to L-malic acid.

All enzyme assays were performed at 30°C with freshly prepared extracts. Total protein concentration was measured by the Lowry method [29], using bovine serum albumin as the standard.

### Transcriptional analysis

For RNA extraction, early-stationary phase cells from flask culture were harvested by centrifugation (8000 rpm at 4°C for 5 min), and stored at-80°C until use. Total RNA was extracted with the RNAprep pure Plant Kit following the manufacturer's instructions. cDNA was synthesized from total RNA by using the PrimeScript^® ^RT reagent kit Perfect Real Time (TaKaRa Biotechnology Co., Ltd.) according to the manufacturer's protocol. Quantitative real-time PCR was performed in a 25 μl (total volume) mixture containing 12.5 μl of SYBR^® ^*Premix Ex Taq*™ II (2×), 400 nmol each of forward and reverse primers, and 2 μl of the cDNA sample. Primers used in the transcriptional analysis are listed in Table 5, and the β*-ACTIN *gene was used as the internal control. Amplification and detection of specific products were performed with a Light Cycler^® ^480 (Roche, Basel, Switzerland). The detection profile used was: incubation at 95°C for 30 s, 40 cycles at 95°C for 5 s, 60°C for 20 s, and 50°C for 30 s. Data analysis was performed using the second derivative method. Each sample was tested in triplicate in a 96-well plate (Bio-Rad). To calculate the relative expression level of the target genes, a relative standard curve method was used. The expression ratio was obtained by dividing the relative expression level of the mutant strain by that of the control strain.

**Table 5:** Primers used in the transcriptional analysis

Primer	Sequences, 5'-3'	Analyzed gene
F(*RoMDH*)	CGCTGCTGGTGGTATTGG	*RoMDH*
R(*RoMDH*)	TGGAGTTGGTGTTGATGTGG	
F(*RoFUM1*)	AAGGCTGCTGCTACTGTC	*RoFUM1*
R(*RoFUM1*)	CACGGTTGGAGATAACTTCG	
F(*PYC2*)	AGAGGTGAGATTCCGATTAG	*PYC2*
R(*PYC2*)	GTCCATTGCCAAGTAAGC	
F(*ACT*)	AGGTATTGCCGAAAGAATGC	β*-ACTIN*
R(*ACT*)	CTTGTGGTGAACGATAGATGG	

## Competing interests

The authors declare that they have no competing interests.

## Authors' contributions

GQX has made contribution to the design of the experiments, the acquisition of data, the analysis and interpretation of data and has contributed to the writing of the manuscript. LML and JC conceived and organized the study and helped to draft the manuscript, and have revised the manuscript. All the authors have read and given their final approval of the version to be published.
